# A Prenatal Case of Arrhythmogenic Right Ventricular Dysplasia

**DOI:** 10.5935/abc.20180022

**Published:** 2018-02

**Authors:** Lilian Maria Lopes, Juliana Torres Pacheco, Regina Schultz, Rossana Pulcineli Vieira Francisco, Marcelo Zugaib

**Affiliations:** 1 Clinica Obstétrica do Hospital das Clínicas da Faculdade de Medicina da Universidade de São Paulo (USP), São Paulo, SP - Brazil; 2 Departamento de Patologia do Hospital das Clínicas da Faculdade de Medicina da Universidade de São Paulo, São Paulo, SP - Brazil

**Keywords:** Arrhythmogenic Right Ventricular Dysplasia, Fetus / echocardiography, Prenatal Care, Pregnancy

## Introduction

Arrhythmogenic right ventricular dysplasia (ARVD) is a heart muscle disorder that is characterized pathologically by fibrofatty replacement of the right (and sometimes left) ventricular myocardium.^1^ In 30-90% of cases, it is an inherited condition, with an autosomal dominant form of transmission.^2^ Disease expression is variable. In this article, we discuss a rare case of fetal ARVD and its difficult prenatal diagnosis, only confirmed at post-natal autopsy.

## Case Report

A healthy 33-year-old woman (gravida 4, para 2) was referred to our tertiary center at 27 weeks' gestation because a previous fetal echocardiography showed an unexplained progression of congestive heart failure after tachyarrhythmia control with digoxin associated with amiodarone. The mother had been seen at another private clinic since 18 weeks' gestation, when the diagnosis was made of a structurally-normal fetal heart, premature atrial contractions, and supraventricular tachycardia with heart rate of 180bpm, treated with digoxin, initially.

**Figure 1:**
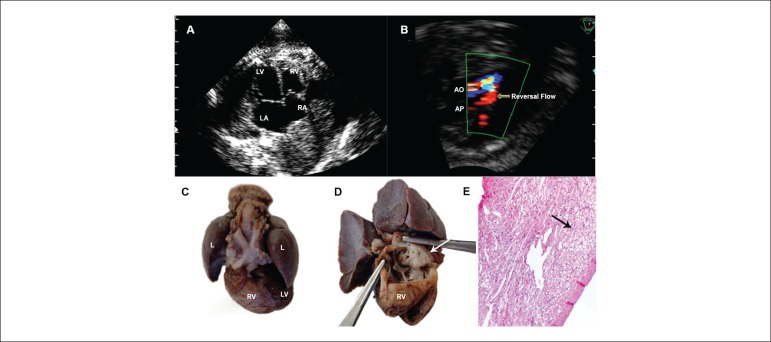
Fetal echocardiography and anatomic features observed at the autopsy. (a) Four-chamber view at 36 weeks showing cardiac enlargement and left atrial dilatation. (b) Three-vessel view showing reversal flow at the ductus arteriosus level (arrow). (c) Heart and lungs with pale, enlarged right ventricle. (d) Right ventricular wall is thin and almost devoid of muscle fibers. (e) A hematoxylin-eosin stain demonstrating absence of myocardial fibers and fibrofatty tissue replacement of the anterior free wall of the right ventricle. RA: right atrium; RV: right ventricle; LA: left atrium; LV: left ventricle; L: lungs.

The patient was then referred to our unit, and fetal echocardiography performed at 27 weeks' gestation showed sinus rhythm with ventricular premature contractions, evidence of global dilatation of all chambers with lower limit shortening fraction of the left ventricle (28%, normal >28%) and a functionally akinetic right ventricle (8%, normal >28%). The presence of a low tricuspid regurgitation velocity of 0.80 m/sec and a reversal flow at the ductus arteriosus level with pulmonary insufficiency suggested a somewhat lower right ventricular systolic pressure (Figure 1). There was fetal hydrops with ascites, pleural effusion, pericardial effusion, and skin edema. The umbilical Doppler indices and ductus venosus flow pattern were within normal ranges, but abnormal umbilical venous pulsations were present. The cardiovascular profile score^3^ was six. Heart failure became worse in the subsequent days, and at 28 weeks' gestation, the patient was hospitalized to investigate other possible causes of fetal heart failure, such as infections, syndromes, and genetic disorders.

The patient's family medical history was unremarkable, and there were no clinical or serological signs of infection. This patient had experienced fetal death at 20 weeks' gestation in her first pregnancy , and her second pregnancy resulted in miscarriage. In the same year, her third pregnancy evolved to biventricular dysfunction, fetal hydrops, and intermittent tachyarrhythmia, again interpreted by another team as supraventricular. Transplacental medication with digoxin was tried at 25 weeks' gestation but did not prevent heart failure progression. At 29 weeks' gestation, the neonate was delivered by cesarean delivery and lived for 14 hours. Fetal autopsy was not performed.

Considering the previous fetal losses, the similarities of the medical history of this pregnancy with the third pregnancy in terms of arrhythmias and the striking finding of right ventricular akinesia in the current fetal echocardiogram, an inherited condition was suspected, and the diagnosis of arrhythmogenic right ventricular dysplasia was considered. At 29 weeks' gestation, diminished fetal movement was observed in the ultrasonographic examination and a cesarean delivery was indicated. A 1,790g male stillbirth was delivered, and histological examination revealed moderate ascites and pleural effusion. Cardiac chambers were greatly dilated, the right ventricular walls were very pale and thin, the left ventricle had an aneurysm at the apex, and the right ventricle showed fibrous tissue and clusters of adipocytes interspersed with myocardial fibers.

## Discussion

Marcus et al.,^4^ described an entity called arrhythmogenic right ventricular dysplasia, characterized by localized deficiency or fibrofatty tissue replacement of the right ventricular myocardium, in the so-called "triangle of dysplasia" (inflow, outflow, and apical regions of the right ventricle), resulting in functional and morphological changes that provide a substrate for both arrhythmias and heart failure,^4^ different from Uhl's disease, which is characterized by a right ventricle wall as thin as a paper and almost devoid of muscle fibers, even though confusion between the two terms has occurred in recent years. Moreover, arrhythmia is more frequent in ARVD, which usually has a right ventricular origin, ranging from frequent premature ventricular contractions (PVCs) to ventricular tachycardia^5^ (VT). Even though our patient had some of the cardinal features of ARVD^6^ (RV dilation/dysfunction and arrhythmia), the diagnosis was only confirmed after the histological findings, as fetal presentation of this disease is rare and literature covering this scope is scarce.^2^

Since ventricular arrhythmias are much more common in ARVD, the diagnosis of supraventricular tachycardia in the third and current pregnancy before referral to our unit was probably misleading, with consequent drug treatment (digoxin) that was not the ideal one. Despite the chosen drug, it seems that in this case the evolution to cardiac failure and death could not be prevented, but we should be very careful when analyzing fetal rhythm, since a correct prenatal diagnosis is crucial for selecting the correct antiarrhythmic treatment and improve chances of survival. This article not only teaches us about the importance of echocardiographic ventricular function evaluation, especially in case of ventricular arrhythmia, but also highlights ARVD as a possible diagnosis in the fetus in early pregnancy.
